# Agricultural activities maintain vigor and plant-based protein intake in older adults with type 2 diabetes: A feasibility study

**DOI:** 10.1016/j.jarlif.2025.100055

**Published:** 2025-12-20

**Authors:** Sachiko Tsukamoto-Kawashima, Kaori Ikeda, Fumika Mano-Usui, Emi Okamura, Aki Kondo, Erina Joo, Eri Maai, Kazusa Nishimura, Tomoyuki Nabeshima, Rihito Takisawa, Yasuki Matsumura, Akira Kitajima, Tohru Tominaga, Ryohei Nakano, Tetsuya Nakazaki, Daisuke Yabe, Nobuya Inagaki

**Affiliations:** a Department of Diabetes, Endocrinology and Nutrition, Kyoto University Graduate School of Medicine, Yoshida Konoe-cho, Sakyo-ku, Kyoto, 606-8501, Japan; b Preemptive Medicine and Lifestyle Related Disease Research Center, Kyoto University Hospital, 54 Shogoin Kawahara-cho, Sakyo-ku, Kyoto, 606-8507, Japan; c Department of Clinical Research Facilitation, Institute for Advancement of Clinical and Translational Science, Kyoto University Hospital, 54 Shogoin Kawahara-cho, Sakyo-ku, Kyoto, 606-8507, Japan; d Research and Development Department, General Incorporated Association Kansai Healthcare Science Informatics, Kyoto, Japan; e Department of Metabolism and Clinical Nutrition, Kyoto University Hospital, 54 Shogoin Kawahara-cho, Sakyo-ku, Kyoto, 606-8507, Japan; f Experimental Farm, Graduate School of Agriculture, Kyoto University, 4-2-1 Shiroyamadai, Kizugawa city, Kyoto, 619-0218, Japan; g Faculty of International Agriculture and Food Studies, Tokyo University of Agriculture, 1-1-1 Sakuragaoka, Setagaya, Tokyo, 156-8502, Japan; h Graduate School of Environmental, Life and Natural Science and Technology, Okayama University, 1-1-1 Tsushima-naka, Kita-ku, Okayama City, Okayama, 700-8530, Japan; i Department of Agriculture, Yamagata University, Tsuruoka, Yamagata, 997-0037, Japan; j Faculty of Agriculture Ryukoku University, 1-5 Yokotani, Oe-cho, Seta, Otsu, Shiga, 520-2194, Japan; k Research Institute for Sustainable Humanosphere, Kyoto University, Gokasho, Uji city, Kyoto, 611-0011, Japan; l Graduate School of Agriculture, Kyoto University, Kitashirakawa Oiwake-cho, Sakyo-ku, Kyoto, 606-8502, Japan; m Office of Institutional Advancement and Communications, Kyoto University, Yoshida-honmachi, Sakyoku, Kyoto, 6068501, Japan; n Medical Research Institute KITANO HOSPITAL, P.I.I.F Tazuke-kofukai, 2-4-20 Ohgimachi, Kita-ku, Osaka, 530-8480, Japan

**Keywords:** Agriculture, Diet, Mood, POMS

## Abstract

**Objectives:**

This study explored the feasibility of agricultural activities for older adults with type 2 diabetes and the psychological and nutritional effects of the activities.

**Methods:**

Nine adults over 65 years of age, capable of performing agricultural activities without assistance were randomly assigned to two groups in a crossover design. Participants engaged in one-hour morning agricultural activities at the Kyoto University farm once a week for 15 weeks (July-November). The Wilcoxon rank-sum test compared Δ values, representing changes from baseline to follow-up, between control and intervention years.

**Results:**

Six participants (three each group) completed the protocol. After the assignment of nine, one participant withdrew, and two others were unable to participate in any agricultural activities due to busy schedules. The procedures were performed safely and no adverse events were reported. Vigor/Activity scores of the Profile of Mood States (POMS-VA) indicated significantly maintained vigor in the intervention year; the median Δ POMS-VA was -13 in the control year and 2 in the intervention year (*P* = 0.01). The Brief-type Self-administered Diet History Questionnaire (BDHQ) suggested that plant-based protein intake was better maintained in the intervention year compared with the control year.

**Conclusions:**

Weekly agricultural activities for older adults with type 2 diabetes showed potential as a therapeutic option, warranting further investigation in a larger and more diverse populations of older patients.

## 1 Introduction

Aging societies are currently experiencing an accelerated increase in the prevalence of diabetes, affecting nearly one in four adults over 65 years worldwide [1,2]. Older adults often experience multiple chronic conditions. Cognitive impairment and mood disorders that develop with age are common risks for poor self-management, which can lead to poor glycemic control [3,4].

Depressive symptoms in older people range from sub-threshold mood disorders to major depression, but these symptoms are underdiagnosed because they are often attributed to normal aging, physical illness, or dementia [3,5]. Depression contributes to a reduced physical activity and quality of life and is a recognized risk factor for hospitalization and mortality in older patients with diabetes. Therapeutic approaches that address psychological as well as metabolic health are essential for comprehensive diabetes care. Interventions targeting mood or motivation, including psychosocial or behavioral programs, have been shown to improve glycemic outcomes in patients with diabetes [6]. Effective diabetes management should address not only blood glucose levels but also self-care and mental health to enable personalized treatment [7,8]. However, little is known about their feasibility or potential role in diabetes care.

We hypothesized that agricultural activities positively influence dietary habits and provide psychological benefits by increasing opportunities for physical activity and social engagement. This small-scale exploratory study assessed the feasibility and potential impact of light agricultural activities on dietary habits and mental benefits in older adults with type 2 diabetes.

## 2 Materials and methods

### 2.1 Procedure

**Fig. 1:**
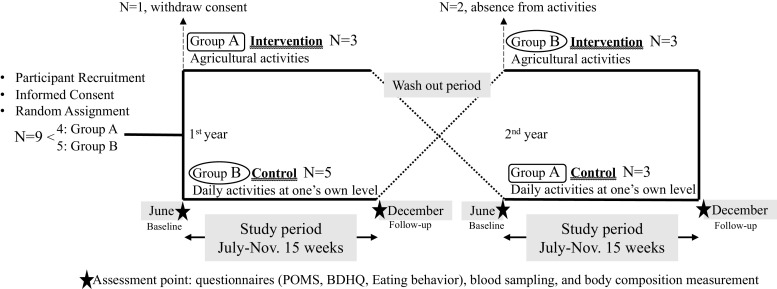
Study Design and Procedure.

The protocol was approved by Kyoto University Graduate School and Faculty of Medicine, Ethics Committee on June 25, 2018 (Approval Number C1384). The study was conducted in the outpatient department of Kyoto University Hospital, according to the principles of the Declaration of Helsinki. This study used a crossover design (Fig. 1).

### 2.2 Participants

Written informed consent was obtained from nine adults over the age of 65 years who were capable of performing agricultural activities without assistance. They were enrolled as eligible participants and randomly assigned, with four allocated to the first-year intervention group (A) and five to the second-year intervention group (B). Each participant engaged in agricultural activities during the intervention year, while maintaining their own level of activities during the control year (Fig. 1).

### 2.3 Intervention

Soybean cultivation and floriculture were selected as agricultural activities because they are sufficiently light for older adults to perform safely. Each session lasted approximately one hour and took place inside a plastic greenhouse to avoid strong sunlight or rain. Air blowers were used in greenhouses to prevent the workspace from becoming too hot. The intervention period was set from July to November, which is the appropriate time for soybean cultivation. During this period, the participants engaged in one-hour activities in the morning once a week for 15 weeks at a farm affiliated with the Kyoto University.

The details of the activities were organized by the medical staff and farm supervisors, with each agricultural procedure guided by technical assistants. The field was cultivated and seeded in July, thinned out, and summer flower seedlings planted in August. In September, the soil around the base of the soybeans was tilled with a hoe, and fall flower seeds were planted between September and October. In the final month of November, the soybeans were harvested and threshed. To assist in conducting the study, weeding and water management were performed by technical assistants as needed on days when the study participants did not visit the farm.

There were no fixed protocols for diabetes pharmacotherapy during the study period. The medications were adjusted according to each patient’s glycemic control status at the discretion of the attending physician.

### 2.4 Measurement

Feasibility and safety were evaluated through clinical measurements and monitoring of adverse events. At study initiation, patient background data including blood glucose, HbA1c, and body composition measured using an InBody720® analyzer (Biospace, Korea) [9] were collected.

Physical activity was assessed using an Omron activity meter (Active style Pro HJA-750C®), worn for one week around each assessment point to estimate daily step counts and exercise (Ex), a unit of physical activity defined by the Japanese Ministry of Health, Labour and Welfare [10]. Ex was calculated by multiplying the Metabolic Equivalent of Task (MET) by the duration of each physical activity in hours, and summing these values across the day to determine daily Ex (MET-hours/day). The seven-day average was used for evaluation.

Psychological well-being was assessed using endocrinological and self-reported measures. Serum cortisol and plasma oxytocin levels were measured as biomarkers of stress. Participants answered self-reported questionnaires, namely the Japanese short version of the Profile of Mood States 2 (POMS), which assesses negative mood, including Total Mood Disturbance (TMD) and the following subcategories: Anger/Hostility (AH), Tension/Anxiety (TA), Depression/Dejection (DD), Fatigue/Inertia (FI), Confusion/Bewilderment (CB), Vigor/Activity (VA), and Friendliness (F). TMD was calculated as the sum of AH, TA, DD, FI, and CB minus VA. The F category is the reference item. T-scores were standardized based on a large reference population, with 50 points representing the mean of that population. For the five negative mood subscales, higher scores indicated stronger negative mood, whereas for the VA subscale, higher scores indicate greater vigor [11].

Diet was assessed using two self-reported questionnaires; the Eating Behavior questionnaire (EB) from Japanese obesity treatment guidelines [12,13] and the Brief-type Self-administered Diet History Questionnaire (BDHQ) [14]. The EB can detect deviations and patterns in eating behavior that can be considered for dietary therapy. The total score and seven subcategory scores were evaluated: ‘Eating as diversion’ ‘Feeling of hunger/satiation’, ‘Eating style’, ‘Motivation to eat’ , ‘Food preferences’, ‘Regularity of eating habits’, ‘and ‘Awareness about weight and constitution’. Higher scores indicate more abnormal eating behavior. The BDHQ is a relatively simple questionnaire designed to assess the amount of nutrients habitually consumed from daily diet (excluding supplements, etc.) for people living in Japan [14].

All indicators were measured at baseline (June) and post-intervention follow-up (December) in each of the two years, with four time points per participant (Fig. 1).

### 2.5 Statistical analysis

The data were analyzed using JMP® 16 (SAS Institute Inc., Cary, NC, USA). To evaluate the changes from before to after the intervention or observation, we calculated the Δ value for each indicator by subtracting the baseline value from the follow-up value. The figure and table show the median Δ values, and significant differences between the control and intervention years were evaluated using the Wilcoxon rank-sum test.

## 3 Results

### 3.1 Characteristic of participants

Nine adults over 65 years of age provided written informed consent and were enrolled. They were randomly assigned to two groups: four to the first-year intervention group (A) and five to the second-year intervention group (B). After the assignment and before the study period, one participant in Group A withdrew consent due to the condition of a comorbid disease. Eight participants participated in the study, and in the intervention year, two participants in Group B were absent from any agricultural activities because of their busy schedules. No adverse health effects were observed in any of the participants. Ultimately, six participants completed the entire study period and were included in the analysis: three females participated in agricultural activities in the first year (Group A) and two males and one female in the second year (Group B). Blood collection data were analyzed for all six participants, whereas questionnaire data were analyzed for only five participants, as one questionnaire was missing in the control year.

At the time of consent, the median age of the six participants was 77 years (range 68–83 years). All patients had type 2 diabetes, with a median disease duration of 25 years (range 9–26 years). The median HbA1c level was 6.4 % (range 5.9–7.7 %), and all participants met the HbA1c targets recommended for older adults [15]. The median body mass index (BMI) was 21.1 kg/m² (range 19.3–32.7 kg/m²). Treatment methods varied from diet and exercise therapy only to oral and injectable medication therapy.

### 3.2 Feasibility and safety

Feasibility was supported by the fact that all scheduled sessions were performed safely without medical assistance. Safety was confirmed through monitoring of clinical indicators (blood glucose, HbA1c, body composition), which showed no adverse deterioration. No hypoglycemia, falls, injuries, or hospitalizations occurred during the study period.

### 3.3 Diabetes control, body composition and physical activity

HbA1c levels showed a slight increase in winter in both years. In the control year, median HbA1c was 6.8 % in June and 7.1 % in December, and in the intervention year, 6.6 % and 7.0 %, respectively. No significant difference in the change in HbA1c levels (Δ HbA1c) between the control year and the intervention year was observed (median Δ HbA1c: 0.25 %, range –0.1 to 1.0; 0.30 %, range –0.2 to 0.7, respectively; *P* = 0.87) (Table 1). No statistically significant differences were observed in Δ BMI or Δ body composition (body fat and skeletal muscle mass) between the two years (Table 1).

**Table 1:** The change in blood indicators and body composition**.**

	**Control year** Median (range)	**Intervention year** Median (range)	***P***
**Blood indicators**			
Δ HbA1c ( %)	0.25 (−0.1∼1.0)	0.3 (−0.2∼0.7)	0.87
Δ Serum cortisol (μg/dl)	1.6 (−5.0∼4.4)	1.3 (−1.0∼2.4)	0.36
Δ Plasma oxytocin (pg/ml)	6.0 (−19.9∼55.4)	−12.0 (−88.4∼−5.6)	0.14
**Body composition by InBody720**			
Δ BMI (Kg/m^2^)	−0.1 (−1.0∼0.4)	−0.2 (−0.8∼1.7)	0.75
Δ Skeletal muscle mass (Kg)	0 (−0.8∼0.1)	−0.1 (−1.0∼0.3)	0.92
Δ Body fat (Kg)	0.9 (−0.5∼2.4)	1.0 (−6.3∼2.9)	0.75
Δ Body fat ( %)	1.5 (−0.7∼4.4)	1.3 (−7.4∼5.2)	0.75

^Δ^ value is the change from baseline to follow-up. *N* = 6, *P* by Wilcoxon rank-sum test.

During the intervention year, two participants underwent minor medication adjustments due to improved glycemic control. During the control year, one participant required medication intensification due to worsening glycemic control. These adjustments were small and clinically appropriate. No participant experienced serious adverse events or hypoglycemia related to the agricultural intervention. No medication changes occurred during the washout period.

Daily step counts and Ex were obtained only from three participants who wore the activity meter at all four assessment points. Daily wearing time ranged from 438 to 828 min per day for each individual, as some participants occasionally forgot to wear the device. The changes in daily step counts varied among individuals. Daily Ex decreased during the winter season, regardless of the intervention. Due to the limited data from only three participants and the variability in wearing time, statistical comparisons were not feasible.

### 3.4 Psychological well-being

Serum cortisol and plasma oxytocin levels, assessed as endocrinological stress indicators, showed no significant changes between the control and intervention years (Table 1).

**Fig. 2:**
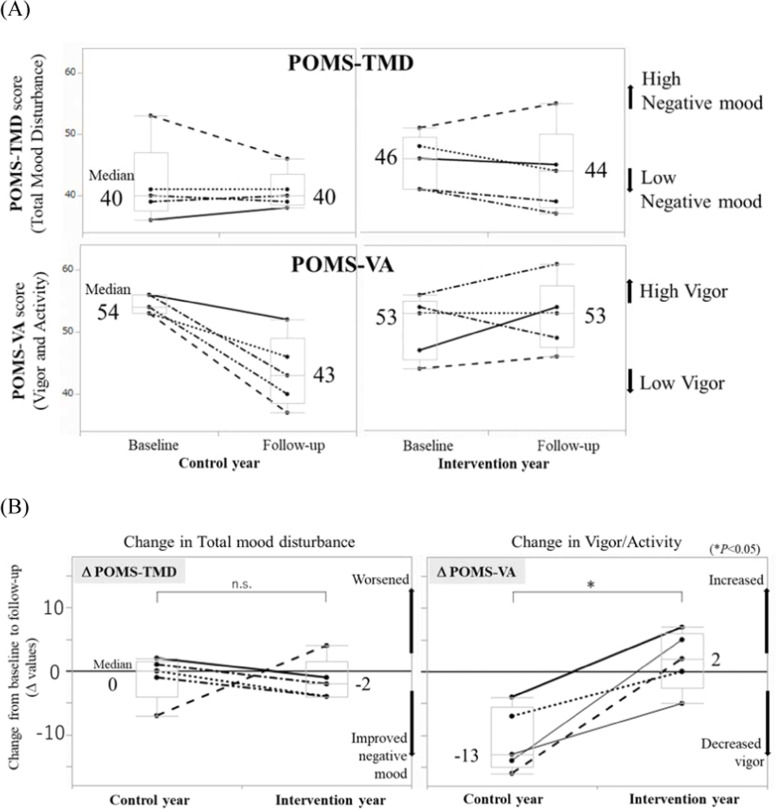
Mood Assessment using the Profile of Mood States (POMS) (*N* = 5). (A) POMS-TMD and -VA scores at each assessment point. (B) Comparison of Δ POMS between the control and intervention years (*P < 0.05 by Wilcoxon rank-sum test).

We plotted the POMS-TMD and POMS-VA scores obtained at baseline and follow-up for the control and intervention years, respectively (Fig. 2A). Among the seven subcategories of POMS, the "Vigor/Activity" subcategory (POMS-VA) showed the largest difference in the median score at the winter follow-up, with 43 and 53 in the control and intervention years, respectively (Fig. 2A). Higher POMS-VA scores indicate greater vigor and sustained vitality. Although the difference in these scores was not statistically significant, the change from baseline to follow-up (Δ POMS-VA) demonstrated significantly greater maintenance of vigor at the winter follow-up in the intervention year, with a median score of –13 (range –16 to –4) in the control year and 2 (range –5 to 7) in the intervention year (*P* = 0.01). The median Δ POMS-TMD was 0 (range –7 to 2) in the control year and –2 (range –4 to 4) in the intervention year, with no significant difference (*P* = 0.53) (Table 2, Fig. 2B).

**Table 2:** The change in score of questionnaires.

	**Control year** Median (range)	**Intervention year** Median (range)	***P***
Δ POMS-TMD (Total mood disturbance)	0 (−7∼2)	−2 (−4∼4)	0.53
Δ POMS-AH (Anger/Hostility)	−4 (−9∼−1)	−4 (−10∼−2)	0.91
Δ POMS-DD (Depression/Dejection)	−2 (−13∼5)	−3 (−5∼16)	0.91
Δ POMS-CB (Confusion/Bewilderment)	0 (−19∼3)	0 (−3∼3)	0.83
Δ POMS-TA (Tension/Anxiety)	−3 (−10∼5)	−2 (−7∼7)	0.46
Δ POMS-FI (Fatigue/Inertia)	−2 (−4∼2)	−3 (−9∼6)	0.91
Δ POMS-VA (Vigor/activity)	−13 (−16∼−4)	2 (−5∼7)	0.01
Δ POMS-F (Friendliness)	−9 (−20∼0)	0 (−11∼6)	0.22
Δ EB ( %)	0.5 (−15.6∼4.5)	−3.2 (−4.9∼1.5)	0.75

^Δ^value is the change from baseline to follow-up. *N* = 5, *P* by Wilcoxon rank-sum test.

### 3.5 Eating behavior

Changes in the Eating Behavior questionnaire score (Δ EB) did not differ significantly between the two years. The median Δ EB was 0.5 (range −15.6 to 4.5) in the control year and –3.2 (range –4.9 to 1.5) in the intervention year (*P* = 0.75) (Table 2).

### 3.6 Nutritional intake

**Fig. 3:**
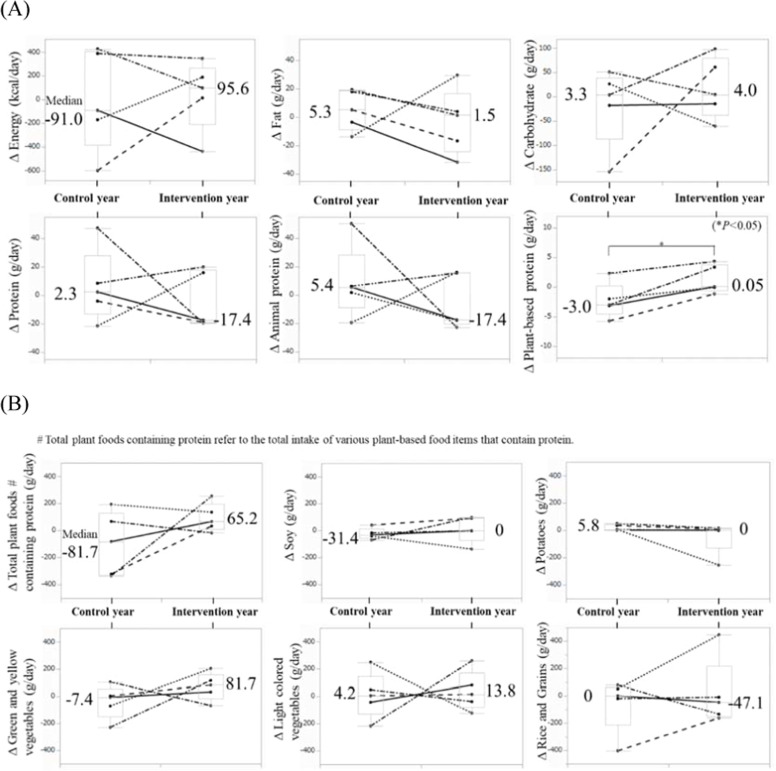
Changes in dietary intake (Δ values) assessed using the Brief-type Self-administered Diet History Questionnaire (*N* = 5, *P < 0.05 by Wilcoxon rank-sum test). (A) Total energy and nutritional intake. (B) Dietary intake of plant foods containing protein.

The daily caloric and nutritional intake, calculated based on the BDHQ, showed no difference in the total energy intake before and after the observation or intervention in either the control or intervention year. The median Δ Energy was –91 kcal/day (range –597 to 425) in the control year and 95.6 kcal/day (range –436 to 343) in the intervention year (*P* = 0.91). Regarding macronutrients, carbohydrate and fat intake remained almost unchanged regardless of the intervention. Protein intake did not differ significantly between the control and intervention years. Among proteins, plant-based protein intake was better maintained in winter follow-up in the intervention year compared to that in the control year. The median Δ Plant-based protein was –3.0 g/day (range –5.8 to 2.3) in the control year and 0.05 g/day (range –1.2 to 4.3) in the intervention year (*P* = 0.04) (Fig. 3A).

Plant foods containing proteins, as defined by the BDHQ, include green and yellow vegetables, light-colored vegetables, soy, potatoes, and rice and grains. For each item, the change in daily intake from baseline to follow-up was compared between the control and intervention years. In the intervention year, the daily intake of plant foods containing protein, particularly green and yellow vegetables, tended to increase more after the intervention compared to that in the control year (Fig. 3B).

## 4 Discussion

This study explored the feasibility of weekly agricultural activities for older adults with type 2 diabetes and their effects on eating behavior, mood and metabolic outcomes. Six participants engaged in a 15-week agricultural program from July to November. Due to the considerable time commitment of weekly one-hour commutes between the hospital and the farm, and the short recruitment period, the number of participants in this study remained small. Nevertheless, all six participants successfully completed the program.

We observed that vigor (POMS-VA), which typically declines in winter, was maintained during the agricultural intervention year. In contrast, the control group experienced a median decrease of 13 points, about 1.3 standard deviations (SD). Although no established minimal clinically important difference exists for the POMS-VA subscale, a systematic review has suggested a 0.5 SD change may represent a minimal important difference for fatigue-related outcomes, [16]. The 1.3 SD decline suggests a clinically significant deterioration in the control group, which the intervention appeared to effectively prevent.

Negative moods, including depressive feelings, are closely linked to poorer self-management and worse glycemic control in individuals with type 2 diabetes [17,18]. Furthermore, such mood often worsens in winter [19,20]. POMS-based studies show that mood deterioration correlates with higher HbA1c levels [21]. Interventions targeting depression, such as medication or psychotherapy, can improve glycemic control [6], emphasizing the clinical importance of mood-enhancing approaches in diabetes care.

Previous reports on gardening and other activity-based interventions consistently demonstrate psychological health improvements, including reduced depressive and anxiety mood states and improved quality of life in both healthy adults and those with chronic diseases, as assessed using established questionnaires other than POMS [[22], [23], [24], [25]]. This difference is notable because prior studies measured mainly depressive mood with single-domain scales, whereas the POMS-TMD is a composite of multiple negative moods. Non-agricultural structured interventions in people with type 2 diabetes, such as occupational and exercise-based programs, have also shown psychological benefits, reducing depressive mood, enhancing self-efficacy, and improving health-related quality of life [24,25]. However, reports evaluating the psychological effects of agricultural interventions specifically in people with diabetes remain limited and are mostly small-scale studies reported in Japanese journals.

The total mood disturbance, a measure of negative mood, showed a slight but non-significant decrease in our study, consistent with prior evidence of psychological benefits from activity-base interventions. Crucially, vigor, a positive mood state that typically declines in winter, was maintained following our agricultural intervention, a finding not clearly demonstrated in earlier studies. Our promising preliminary findings on psychological function suggest that agricultural activities merit future efficacy studies to fully determine their role as a mood-supportive strategy in diabetes care.

We observed no significant improvements in glycemic control, likely due to medication adjustments individually based on clinical judgment. In the intervention year, two participants reduced medications due to improved glycemic control, while one in the control year required a slight increase due to worsening glycemic control.

Dietary assessment revealed no significant changes in overall energy or major nutrient intake. However, plant-based protein intake was maintained throughout the winter follow-up after participating in agricultural activities. Among Japanese adults, seasonal variation in plant-based protein intake is reported, with lower intake in autumn and winter and higher intake in spring and summer [26]; the observed maintenance of intake in the intervention year suggests that agricultural activities may help prevent the seasonal decline in plant-based protein intake typically observed in the general population.

Nutritional therapy in older adults should be carefully managed, as dietary restrictions aimed at improving glycemic control can accelerate age-related muscle loss [27]. Plant-based proteins generally contain less fat than animal proteins and provide more dietary fiber. In older adults with type 2 diabetes, replacing animal proteins with plant-based proteins has been associated with improved glycemic control [28]. Total protein intake is positively correlated with skeletal muscle mass in elderly patients with type 2 diabetes, and plant-based proteins do not contribute as much to weight gain as animal-based proteins [29].

The main limitations of this study include the small sample size (only six participants completing the protocol) and the high selectivity of the participants (older adults meeting specific criteria for health, motivation, and scheduling flexibility). Consequently, this limited statistical power, restricted generalizability, and likely introduced selection bias. Furthermore, since the intervention occurred during summer and fall and the follow-up was in winter, some observed changes may reflect seasonal effects rather than the intervention alone. However, the use of crossover design, where the intervention and control periods occurred in the same seasons of different years, mitigated the influence of seasonal variations on the comparisons. Although the findings should be interpreted with caution, the study provides preliminary evidence that weekly agricultural activities are feasible and safe for older adults with type 2 diabetes, supporting the need for larger trials.

## 5 Conclusions

Weekly agricultural activities for older adults with type 2 diabetes have shown potential as therapeutic options, warranting further investigation in larger and more diverse populations of older patients.

## Fundings

This study was supported by JSPS KAKENHI Grant Number JP17KT0147.

## Data sharing statement

The data analyzed in this study are not publicly available but may be obtained from the corresponding author upon reasonable request. Interested researchers may contact the corresponding author via email to request access to de-identified individual data or relevant supporting materials.

## Declaration of the use of generative AI

Generative AI tools were used exclusively for English language editing. This manuscript underwent English language editing using the paid version of Paperpal, an academic writing–specific editing tool developed by Editage. The tools did not generate any scientific content, interpretations, or analytical results. All substantive intellectual contributions were made by the authors.

## CRediT authorship contribution statement

**Sachiko Tsukamoto-Kawashima:** Writing – review & editing, Writing – original draft, Methodology, Investigation, Formal analysis, Data curation. **Kaori Ikeda:** Writing – review & editing, Supervision, Project administration, Methodology, Investigation, Funding acquisition, Conceptualization. **Fumika Mano-Usui:** Investigation. **Emi Okamura:** Investigation. **Aki Kondo:** Investigation. **Erina Joo:** Investigation. **Eri Maai:** Resources. **Kazusa Nishimura:** Resources. **Tomoyuki Nabeshima:** Resources. **Rihito Takisawa:** Resources. **Yasuki Matsumura:** Resources. **Akira Kitajima:** Resources. **Tohru Tominaga:** Resources. **Ryohei Nakano:** Resources, Project administration. **Tetsuya Nakazaki:** Supervision, Project administration, Methodology, Investigation, Conceptualization. **Daisuke Yabe:** Writing – review & editing. **Nobuya Inagaki:** Supervision.

## Declaration of competing interest

The authors declare the following financial interests/personal relationships which may be considered as potential competing interests:

Kaori Ikeda reports financial support was provided by JSPS KAKENHI Grant Number JP17KT0147. Nobuya Inagaki reports a relationship with Sumitomo Pharma Co Ltd that includes: funding grants and speaking and lecture fees. Nobuya Inagaki reports a relationship with Nippon Boehringer Ingelheim that includes: funding grants and speaking and lecture fees. Nobuya Inagaki reports a relationship with Mitsubishi Tanabe Pharma Corporation that includes: funding grants and speaking and lecture fees. Nobuya Inagaki reports a relationship with Asken that includes: funding grants and speaking and lecture fees. Nobuya Inagaki reports a relationship with Novo Nordisk Pharma Co Ltd that includes: speaking and lecture fees. Nobuya Inagaki reports a relationship with Eli Lilly Japan KK that includes: speaking and lecture fees. Nobuya Inagaki reports a relationship with Ono Pharmaceutical Co Ltd that includes: speaking and lecture fees. Nobuya Inagaki reports a relationship with Teijin Pharma that includes: speaking and lecture fees. Nobuya Inagaki reports a relationship with Kowa Company Ltd that includes: speaking and lecture fees. Nobuya Inagaki reports a relationship with Sanwa Kagaku Kenkyusho Co Ltd that includes: speaking and lecture fees. Nobuya Inagaki reports a relationship with Sanofi that includes: speaking and lecture fees. Nobuya Inagaki reports a relationship with MSD KK that includes: speaking and lecture fees. Nobuya Inagaki reports a relationship with Kyowa Kirin that includes: speaking and lecture fees. Nobuya Inagaki reports a relationship with Kissei Pharmaceutical Co Ltd that includes: speaking and lecture fees. Daisuke Yabe reports a relationship with Novo Nordisk Pharma Co Ltd that includes: speaking and lecture fees. Daisuke Yabe reports a relationship with Eli Lilly Japan KK that includes: speaking and lecture fees. Daisuke Yabe reports a relationship with Nippon Boehringer Ingelheim Co Ltd that includes: speaking and lecture fees. If there are other authors, they declare that they have no known competing financial interests or personal relationships that could have appeared to influence the work reported in this paper.
